# Resource Wars and Conflict Ivory: The Impact of Civil Conflict on Elephants in the Democratic Republic of Congo - The Case of the Okapi Reserve

**DOI:** 10.1371/journal.pone.0027129

**Published:** 2011-11-09

**Authors:** Rene L. Beyers, John A. Hart, Anthony R. E. Sinclair, Falk Grossmann, Brian Klinkenberg, Simeon Dino

**Affiliations:** 1 Beatty Biodiversity Centre, University of British Columbia, Vancouver, British Columbia, Canada; 2 Tshuapa-Lomami-Lualaba Project (TL2), Kinshasa-Gombe, Democratic Republic of Congo; 3 Wildlife Conservation Society, Africa Programs, New York, New York, United States of America; 4 Department of Geography, University of British Columbia, Vancouver, British Columbia, Canada; University of Pretoria, South Africa

## Abstract

Human conflict generally has substantial negative impacts on wildlife and conservation. The recent civil war (1995-2006) in the Democratic Republic of Congo (DRC) resulted in a significant loss of wildlife, including elephants, due to institutional collapse, lawlessness and unbridled exploitation of natural resources such as minerals, wood, ivory and bushmeat. We used data from distance sampling surveys conducted before and after the war in a protected forest, the Okapi Faunal Reserve, to document changes in elephant abundance and distribution. We employed Generalized Additive Models to relate changes in elephant distribution to human and environmental factors. Populations declined by nearly fifty percent coinciding with a major increase in elephant poaching as indicated by reports of ivory trade during the war. Our results suggest that humans influenced elephant distribution far more than habitat, both before and after the war, but post-war models explained more of the variation. Elephant abundance declined more, closer to the park boundary and to areas of intense human activity. After the war, elephant densities were relatively higher in the centre of the park where they were better protected, suggesting that this area may have acted as a refuge. In other sites in Eastern DRC, where no protection was provided, elephants were even more decimated. Post-war dynamics, such as weakened institutions, human movements and availability of weapons, continue to affect elephants. Survival of remaining populations and recovery will be determined by these persistent factors and by new threats associated with growing human populations and exploitation of natural resources. Prioritizing wildlife protection, curbing illegal trade in ivory and bushmeat, and strengthening national institutions and organizations in charge of conservation will be crucial to counter these threats.

## Introduction

There is now overwhelming evidence that wars and other forms of human conflict disturb ecosystems and cause the loss of biodiversity. This loss is particularly acute with large species [1], [2]. The African elephant (*Loxidonta africana*) is therefore one of the most vulnerable to human conflict as it requires large areas of suitable habitat, and so suffers from habitat loss. Furthermore they are prime targets for ivory and meat hunters.

In the Democratic Republic of Congo (DRC) all elephant populations suffered during the war of 1995 - 2006. Displaced peoples resulted in significant habitat loss, as occurred in the Virunga National Park, DRC, where an area of 300 km^2^ was deforested during the refugee crisis following the genocide in Rwanda in 1994 [3], [4]. Populations of elephants were severely reduced by armed militias who competed to secure control and monetary off-take of easily extractable natural resources, such as gold, diamonds, and mineral ores, that could be extracted by low input artisanal methods. Saw-wood, charcoal and fisheries were also targets of control and conflict. Key resources included ivory and bushmeat, and African elephants were the most important of these targeted species.

DRC's conflict led to widespread lawlessness. Government institutions were disrupted or taken apart, or oriented to facilitate illegal extraction and taxation (such as the national police and military). Institutions such as the national parks service, whose mandate is the protection and control of natural resources, were the focus of attack and harassment. Thus, the collapse of wildlife conservation and enforcement during the conflict was profound. Staff ceased normal operations or moved out of protected areas, and many were killed. Hunting increased and was partly linked to the proliferation of small arms. Militias and military occupied protected areas. The exploitation of elephants for ivory and meat was used to provision insurgents or the military, and to generate revenue to fund further expansion of resource takeovers [5].

**Figure 1:**
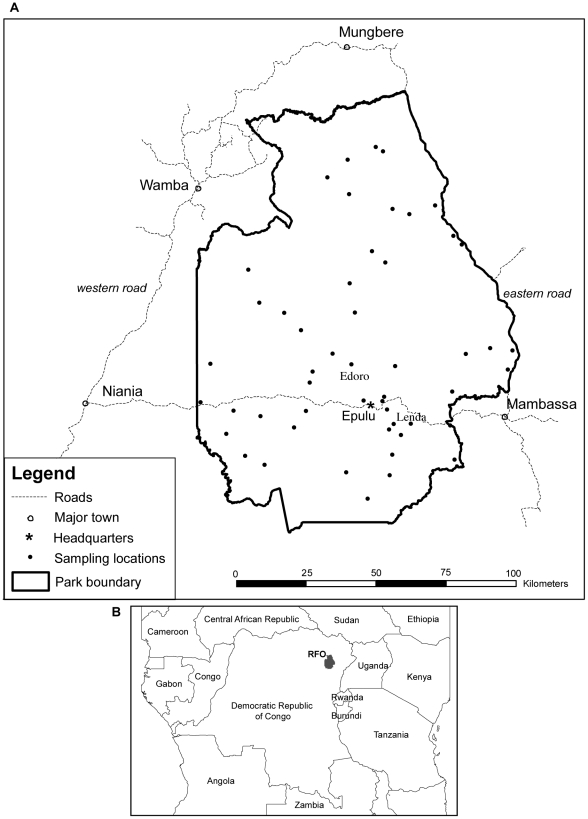
Map of the Okapi Faunal Reserve with sampling locations. A. Boundaries of the reserve, roads, main towns, park headquarters and sampling locations. B. Geographic location in Central Africa.

In the context of this widespread and profound impact of human killing of elephants, we ask: what were the effects of the conflict on elephants, and whether legally gazetted and demarcated reserves continued to provide protection or whether the conservation system broke down entirely? To address these questions we focus on forest elephants (*Loxodonta africana cyclotis*) in the Okapi Faunal Reserve (RFO) in eastern DRC (Figure 1). This was one the largest forest elephant populations in the region at the outset of the conflict in 1996. Numbers and distribution of this population were better documented than that for any other population of forest elephants. In addition, we monitored what happened to the elephants as a consequence of the conflict, something that was not possible in other forested areas.

First, we present a history of what happened to the RFO and its elephants before and during the conflict. We give an overview of reported elephant poaching and ivory seizures.

Second, we evaluate changes in the numbers of elephants in the reserve. The RFO, as a forest reserve, presents a unique opportunity in DRC in that wildlife surveys had been carried out from 1994–1995 before the onset of the conflict and immediately after the conflict from 2005–2007 using exactly the same transect sampling design. This allows comparisons of pre- and post conflict population densities. We also look at changes in their distribution.

Third, we assess the role of human activities and habitat in the distribution of elephants and evaluate changes in these relationships before and after the war. We predict human influence to be higher during and after the war. We test hypotheses relating proxies for human impact and habitat to elephant densities (Table 1). We expect to find fewer elephants in more accessible areas and use distance from the nearest road as a proxy for human access. We use covariates such as distance from human settlements and the distance to and size of deforested areas (as an index of deforestation) as proxies for human density and activity. We expect more elephants and smaller declines in areas that were better protected. As indicators of protection, we include covariates such as distance from the reserve boundary and distance from protection bases. We employ spatial modeling techniques using Generalized Additive Models and line transect density estimates, details of which are presented in the methods section later.

**Table 1:** Candidate covariates included in the spatial models for 1995 and 2006.

Name	Covariate	Source	Hypotheses
**HUMAN-RELATED COVARIATES**			
Roads	Distance from the nearest road	Landsat ETM image (1)	Roads provide access to poachers and elephant densities are lower closer to the nearest road.
Villages	Distance from the nearest village	GPS waypoints	Elephants are less abundant near human habitation and densities are lower closer to the nearest village.
Major towns	Distance from the nearest major town	GPS waypoints	Big towns have proportionally more impact on elephants than smaller settlements and elephant densities are lower closer to the nearest larger town.
Park	Distance from the park boundary	CARPE database (2)	The park boundary acts as a protective barrier against poachers and elephant densities are higher further inside the park.
Park headquarters at Epulu	Distance from the nearest guard post	GPS waypoints	Park headquarters provides protection to wildlife and there is a negative relationship between elephant densities and distance to the nearest guard post.
Deforestation index	Composite index of deforestation extent and distance from each transect to all deforestation sites on a predefined grid	Landsat and Modis images, GIS (3)	Elephant abundance is negatively associated with proximity to and extent of deforestation as a proxy for human population density
**HABITAT-RELATED COVARIATES**			
Slope	Average slope within a 100 meter buffer along transect	DEM constucted from SRTM (4)	Slope influences abundance of elephants either directly or through different types of vegetation that are associated with a different topography.
Habitat	Ecozones	Digitized from Landsat satellite images and field data (5)	Elephants prefer certain habitats over others.

(1) Digitized from Landsat images by the Department of Geography, University of Ghent (http://geoweb.ugent.be/sygiap/).

(2) Database of the Central African Regional Program for the Environment (CARPE, http://carpe.umd.edu/).

(3) Carpe Decadal Forest Change Mapping project (CARPE Decadal Forest Change Mapping (DFCM) Project, http://carpe.umd.edu/resources/dfcm) and South Dakota State University (Erik Lindquist).

(4) Space Shuttle Radar Topography Mission (SRT Seamless Data Distribution System, Earth Resources Observation and Science (EROS), http://seamless.usgs.gov).

(5) Obtained from Landsat 7 images (2002) and field data from transects and classified into the following ‘ecozone’ categories: mixed hill forest, rocky outcrops (inselbergs), savanna-forest ecotone, mixed forest, mono-dominant forest consisting of *Gilbertiodendron dewrevei*, swamp forest and non-forested area.

## Results

### Chronology of the conflict and elephant poaching in the RFO

Poaching of elephants in DRC, including the RFO, was rampant from the late 1970′s to the early 1980′s [6]. It stopped almost completely in the RFO after the CITES ban on ivory trade in 1989 [7]. Between the late 1980′s and 1996, there was little poaching in the reserve.

**Figure 2:**
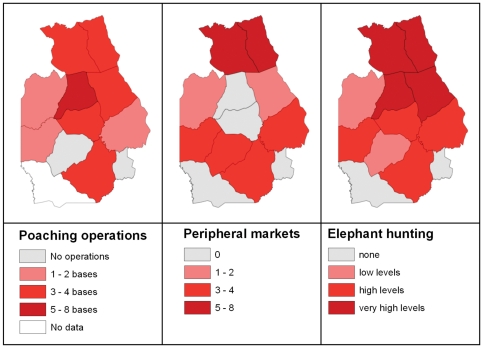
Poaching operations, bushmeat and ivory markets and poaching intensity in the RFO, 2002–2003. Number of poaching operations observed (left map), number of bushmeat markets observed (middle map) and estimation of elephant hunting intensity (right map).

The period of the latest conflict in the area began in 1996 (Figure S1), when military and rebel factions moved into the area, looted park headquarters, disarmed park guards, brought in hunters, and opened markets around the reserve for bushmeat and ivory. These militias were replaced by Uganda-backed rebels in 1999. The killing of elephants was widespread in 2000, and military deserters set up large poaching camps to the southeast, southwest and west of the reserve, as well as inside the northeastern part [5]. During a five month anti-poaching operation (“Operation Tango”), through a collaborative effort of ICCN staff, military, paramilitary and NGO's, 117 kg of ivory (Table 2) and 215 kg of elephant meat was recovered, and 20 poachers were apprehended [8]. Elephants in the region were killed not just for ivory but also to feed armed forces between Bunia and Kisangani [9]. The worst of the killing happened between 2002 and 2004, when rebel militias clashed in areas of high elephant density. In 2002, data from surveys of ivory transporters and local markets, and undercover operations, indicated that 6.5 tons of ivory had left the reserve and adjacent areas over a period of 12 months (Table 2). Undercover operations by ICCN in 2004 led to the discovery of an estimated 14.3 tons of ivory. Based on reports from locations of poaching bases and peripheral meat and ivory markets, we estimated that in 2002–2003, hunting was particularly severe in the northern part of the park but relatively low in the central core north and south of the road traversing the reserve (Figure 1, 2).

**Table 2:** Reported ivory in the RFO during the conflict.

Source	Location	Evidence	Period over which data was collected (months)	Period when collected	Ivory (kg)	Percent of total
Operation Tango [9]	Whole reserve	Seizures	5	2000	117	0.5
ICCN (1)			12	2002	6570	27.7
	Settlements bordering Reserve	Survey transporters	1.5	2002	50	
	Isiro	Local market survey	0.5	2002	20	
	Mambasa	Undercover report	7	2002	3700	
	Apodo	Undercover report	4	2002	2800	
ICCN (2)			7	June–Dec 2004	17000	71.8
	East side of reserve	Undercover report	4	June–Sep 2004	8386	
	East side of reserve	Undercover report	3	Oct–Dec 2004	8614	
TOTAL					23687	

(1) Hart, JA (2003) Conflict Ivory: Elephant Poaching and Ivory Traffic in the Ituri forest during the Congolese Civil War: 1996–2004. A Collaborative Documentation: ICCN, WCS, MIKE and Gilman Intl Conservation, US Fish and Wildlife Service. Unpublished presentation.

(2) Apopo CA (2004) Rapport sur le braconnage à l'Eléphant et sur la commerce de l'ivoire dans et à la_périphérie de la Réserve de Faune à Okapis. Inventory and Monitoring Unit, Rapport No 3, December 2004, Widlife Conservation Society, Democratic Republic of Congo, 33 p.

There was no recorded elephant poaching in the RFO in 2006 following recovery of the site by ICCN with the support of military, the FARDC (Forces Armées de la République Démocratique du Congo). There were only 12 reports of poaching from 2007 through 2008. In 2009 there was again an upsurge in poaching, led by FARDC, with 8 cases reported and 36 elephants reported killed (John Hart, personal communication).

### Declines in elephant densities and changes in distribution

We used a z-test to compare elephant dung density across the reserve before and after the conflict, dung being our index of elephant numbers. Dung density declined by 48% (z = 1.978, p = 0.024) from 4.09 to 2.13 dung piles per ha (Table 3). We used conversion factors to obtain elephant density from dung decay rates from a previous study in the RFO [7] and from dung defecation rates, which were measured elsewhere [10]. Using a defecation rate of 19.77 dung piles per day and a mean estimated dung decay rate of 44 days, and assuming that dung decay rates were similar during both survey periods, the observed decline in dung abundance corresponded to a decline of actual animal densities from 0.47 to 0.24 elephants per km^2^. If we consider this estimate to be representative for the entire reserve, the loss in elephants amounted to 3151 animals in the last decade, from 6439 individuals to a post-war population of 3288. These absolute figures should be treated with caution because defecation rates may be different in the RFO and seasonal, climatological and habitat related factors that affect decay rates of dung were not taken into account [11], [12].

**Table 3:** Survey effort, encounter rates and elephant dung densities in the RFO from the data that were used for spatial models.

Survey	Samples	Total effort (km)	No obs.	n/L(per km)	CV (n/L)	ESW (m)	D (per ha)	CV (D)	CI
1995	51	280	460	1.64	18.63	2.01	4.09	19.07	2.83–5.93
2006	51	280	286	1.02	28.08	2.40	2.13	28.70	1.22–3.70

CV (n/L)  =  coefficient of variation for dung encounter rates (dung piles per km), ESW  =  effective strip width, D  =  dung density per hectare, CV (D)  =  coefficient of variation for dung density per hectare, CI  =  confidence interval for dung density per hectare.

**Figure 3:**
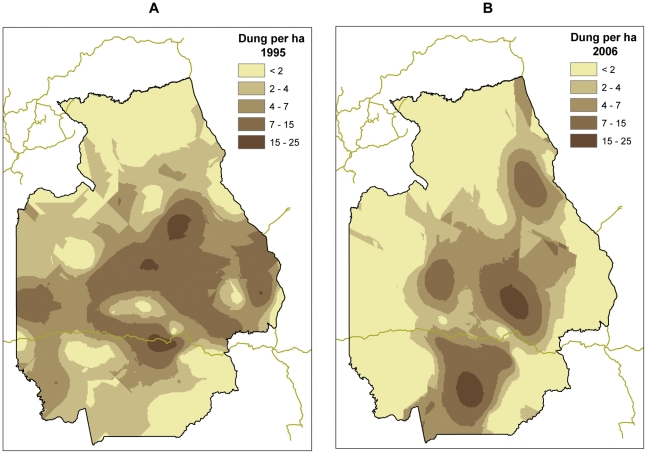
Elephant density surface maps for 1995 and 2006 derived using Ordinary Kriging. A. Dung density surface (dung per hectare) in 1995. B. Dung density surface (dung per hectare) in 2006.

The distribution of elephant dung densities in 1995 and 2006 is visually presented on the kriging maps (Figure 3). Hotspot analysis of dung densities (Getis-Ord Gi*, see methods) in 1995 showed hotspots significantly higher than expected (z score >1,96, p<0.05) in the center (2) around Epulu (2) and near the eastern (2) and western boundary (2) of the reserve. In 2006, hotspot analysis showed hotspots with significantly higher densities (z>1.96, p<0.05) in the south (1) and north of the road that traversed the reserve (1), both within the zone of protection by rangers and higher security during the conflict. Two high-density hotspots within the same area, north and south of the road, were close to significance (z = 1.873, p = 0.061). There was also one hotspot of higher densities in the northeast, which is unexplained.

Of the 52 transect locations, dung densities declined in 28, remained the same on 10 and increased on 13, which is significantly different from what we would expect from random change (Chi square 10.9412, p = 0.004). As mentioned above, the magnitude of the decline in dung density across all transects was also significant (z = 1.978, p = 0.024). Of the 9 locations where there was an increase in dung density of more than 0.1 dung piles per km^2^, 7 occurred in the zone of higher protection and 2 were in the northeast.

### Pre-war spatial models

**Figure 4:**
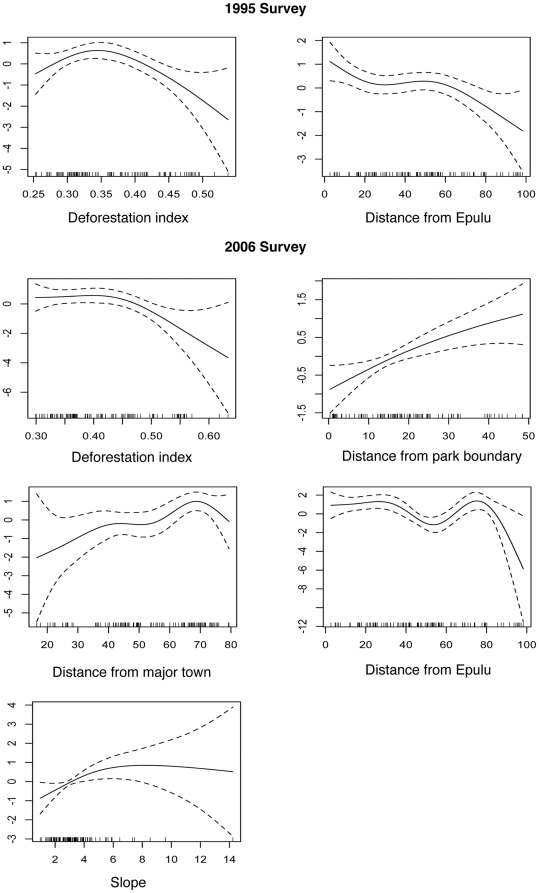
Gam plots of the effect of each smoothed variable on estimated dung densities. The gam plots show the nature of the modeled relationships between the smoothed predictor and the dependent variable. The effect of the predictor on the dependent variable is shown on the y-axis for different values of the predictor (x-axis). Estimates are shown by the solid line and 95% confidence intervals by the dashed lines. A rug plot just above the X-axis indicates the density of observations.

The spatial models (General Additive Models) confirmed most of our *a priori* formulated hypotheses and were consistent with the patterns observed on the spatially-interpolated (kriging) maps. Humans influenced elephant distributions in both time periods, but their impact appeared to be far greater during and after the war than before the conflict. In 1995 deforestation in the buffer zone around the reserve was the best predictor of elephant distribution and explained 15% of the deviance in a univariate model (Table 4). Densities were lower close to areas with more active deforestation (Figure 4). Elephants were more abundant closer to park headquarters in Epulu, but their numbers remained stable between 20 and 60 km from Epulu before dropping off at the periphery of the reserve. Their density increased with distance from the park boundary, but only 6% of the variance was explained by this predictor. Contrary to our hypothesis, distance from the road was not a significant covariate. The only significant habitat covariate was hill forest, which on its own explained only 6% of the variance.

**Table 4:** GAM's of elephant dung densities in the Okapi reserve in 1995 and 2006.

Models and covariates	Deviance explained (%)	GCV score
**1995 univariate models**		
deforestation index *	15.20	8.147
distance from Epulu headquarters (L-) *	13.20	8.437
hill forest (L+) *	6.00	8.785
distance from park boundary *	5.59	8.824
distance from nearest major town	1.95	9.164
slope (L-)	0.40	9.308
distance from the nearest road	0.03	9.174
**1995**‘**human covariates only**’ **model**	17.20	8.056
**1995 minimal adequate model including habitat**	21.67	7.941
**2006 univariate models**		
deforestation index *	11.9	7.471
distance from Epulu headquarters *	24.60	6.710
distance from nearest major town *	16.90	7.268
distance from park boundary *	9.93	7.509
swamp forest (L+) *	8.57	7.566
slope *	7.12	7.825
distance from the nearest road	3.26	8.257
**2006**‘**human covariates only’ model**	70.10	3.709
**2006 minimal adequate model including habitat**	72.20	3.494

“(L)” denotes a linear term and the “+” or “-” sign denotes a positive correlation or a negative correlation with the linear term. Other variables are smoothed and the nature of their relationship with the predictor is dependent on the value of the predictor and is shown on their respective gam plots (figure 4) * denotes a significant difference with the null model. Only significant habitat variables are given.

A multivariate minimal adequate model with human-related covariates explained a very modest 17.2% of the variation and included deforestation index and distance from Epulu as significant covariates. A model with human and habitat covariates was marginally better, explaining only 21.7% of the variance.

### Post-war spatial models

Elephant density patterns across the reserve and density relationships with covariates changed substantially after the conflict. Models with human-related covariates explained much more variation (70.1%) than in the first survey period (Table 4). The best predictor was distance from headquarters in Epulu (24.6% of deviance explained). Elephants were more abundant up to 40 km from Epulu (Figure 4). Abundance dropped after that but there was an increase again at 80 km, which corresponded to the distance of the sampling location with higher densities in the northeast of the reserve. Distance from the nearest major town was the second best predictor (16.9% deviance explained), and elephants became more abundant with increasing distances from the nearest major settlement. Elephant density increased gradually further away from the park boundary, but as in the 1995 model, distance from the park boundary was a relatively weak predictor (9.9% of deviance explained). There was more evidence of elephants on transects that were further away from areas of high deforestation, indicating a relationship with human activity. Distance from nearest road, however, did not have a significant effect on dung densities in a univariate model.

Contrary to our expectations, elephants increased on steeper slopes, although the effect was small (7.1% explained deviance). Elephant abundance was higher in swamp forest, which was the only habitat variable with a significant relationship.

A multivariate minimal adequate model with only human variables retained the deforestation index, distance from the nearest major town, distance from Epulu, distance from the reserve boundary and distance from the nearest road as significant covariates. The model accounted for 70.1% of the variation, which indicates a strong increase of human impact on elephants compared to that before the conflict. There was a negligible improvement of 2% with a model including habitat types and slope, suggesting that human impact was far more important in determining elephant distribution than ecological factors.

## Discussion

### Conflict and elephants in DRC

During the last 50 years Africa has been plagued by a large number of armed conflicts. Most of these conflicts were internal to the countries concerned and included civil wars. Many occurred in countries with a rich biodiversity and over 80% occurred fully or partially in biodiversity hotspots [2]. At least half of the conflict zones included forests and, in Africa, conflicts have affected up to two thirds of forested lands [13].

Impact of conflict can vary. Conflicts can be beneficial for wildlife, notably where opposing armies enforce no-go zones with negligible human use and occupation. A classic example is the Demilitarized Zone between North and South Korea where wildlife populations have boomed [2]. However, in most cases, such as in DRC, conflicts inflict substantial damage to the environment, protected areas and wildlife [1]. Impacts can be both direct and indirect. Direct impacts can be a result of activities of military and militias, indirect impacts can be caused by weakening protection and the rule of law, and the increased availability of arms. Furthermore, processes affecting the environment already underway may be accelerated or enhanced by conflict.

In the RFO, direct and indirect impacting factors combined to wreak havoc on the elephant population. Despite the protected status of the reserve, up to 50% of the population, or perhaps as many as 3300 animals, have been lost. It was estimated that at least 23 tons of ivory was taken out of the reserve and its surroundings. Assuming an average 6.9 kg of ivory per elephant [14], this corresponds to 3434 dead elephants, close to the estimated decline in the elephant population between 1995 and 2007. However, some of the documented ivory came from surrounding areas and not all killing was reported either.

Intelligence information obtained from businessmen, ivory dealers and journalists indicated that ivory from northeastern DRC was shipped to Uganda and the Central African Republic CAR (Apopo CA. Rapport sur le braconnage à l'Eléphant et sur la commerce de l'ivoire dans et à la_périphérie de la Réserve de Faune à Okapis. Inventory and Monitoring Unit, Rapport No 3, December 2004, Widlife Conservation Society, Democratic Republic of Congo). Both rebel commanders and businessmen were implicated in trafficking. Hunter et al. [14] estimated that about 4000 elephants were needed each year to supply both the illegal international (mainly Asian) and the internal African ivory market, and most of this ivory was believed to have come from Central Africa, especially eastern DRC. The result of our analysis, combined with the information on ivory, suggests that the Ituri region was an important global source of ivory from 2002 to 2004.

### Comparison with other areas in Eastern DRC

Essentially all of DRC's elephant populations suffered during the war. Table 5 presents four other protected areas alongside the RFO where changes in elephant populations were documented with varying degrees of accuracy and precision. There are two elephant subspecies that occur in DRC, the African forest elephant (*Loxodonta africana cyclotis*) and the African savanna elephant (*Loxodonta africana africana*). There is now strong evidence based on DNA research that these two subspecies should be treated as two different species, *Loxodonta cyclotis* and *Loxodonta africana* respectively [15].

**Table 5:** Elephant population declines in the RFO compared to other sites in DRC.

Elephant Range	Historical record (pre-1980)	Before war (1986–1996)	Civil War (1996–2003)	Post-war Anarchy (2003–2009)	References
Okapi Forest Reserve (RFO)	N.D.	6,439	N.D.	3,288	this analysis
Garamba National Park (forest, savanna)	22,670	11,175	5,983	3,696 (1)	^(2–4)^
Maiko National Park (forest) (5)	N.D.	6000	N.D.	1000–3000	[46, (6)]
Kahuzi Biega NP – upland forest	N.D.	±800	±20	±20	[46]
Kahuzi Biega NP – lowland forest	N.D.	3,720	N.D.	No sign (7)	[16,46, (8)]
Virunga National Park (savanna)	2,900	469	286	300–350	[46, ^(9–10)^]

(1) the post-war survey in 2007 covered the southern core zone of the park only (2567 km^2^) as this was the area where remaining populations of elephants were concentrated. Very few elephants were reported outside this area.

(2) de Merode E, Bila I, Telo J, Panziama G (2005) An aerial reconnaissance of Garamba National park with a focus on northern white rhinoceros. Technical report to ICCN and the European Union Further technical input from ACF and WWF-CARPO staff.

(3) Emslie RH, Reid C, Tello J (2006) Report on the different target species counted and evidence of poaching activity recorded during aerial and ground surveys undertaken in southern Garamba National Park and adjoining Domaine de Chasse Gangala Na Bodio, DR Congo 17th–30th March 2006. ICCN, AP, IUCN-SSC, UNESCO.

(4) Hillman-Smith K, Atalia M, Likango M, Smith F, Ndey A, et al. (1995) General aerial count 1995 and evaluation of the status and trends of the ecosystem. Garamba National Park Project Report. Unpublished.

(5) estimates are informed guesses based on recce surveys in a sub-area of the protected area. Current populations may be lower. Surveys in sub-area in 2005 indicate a 150 times lower encounter rate of elephant dung than in the same area in 1992.

(6) Nixon SC A Preliminary Survey of the Maiko National Park Southern Sector and Adjacent Forests, January-May 2005. Dian Fossey Gorilla Fund International, unpublished field report.

(7) this was not an exhaustive survey due to continued rebel presence, but the lack of elephant signs is ominous.

(8) Hart J, Carbo M, Amsini F, Grossmann F, Kibambe C (2007) Parc National de Kahuzi-Biega, Secteur de Basse Altitude: Inventaire préliminaire de la grande faune avec une évaluation de l'impact des activités humaines et la situation sécuritaire 2004–2007. Inventory and Monitoring Unit, Rapport No7, Novembre 2007, Widlife Conservation Society, Democratic Republic of Congo.

(9) Kujirakwinja D, Plumptre A, Moyer D, Mushemzi N (2006) Parc National des Virunga. Recensement aerien des grands mammifères. Institut Congolais pour la Conservation de la Nature, Wildlife Conservation Society, US Fish and Wildlife Service.

(10) Matunguru J (2007) Rapport de mission sur le suivi des éléphants effectuée à Kabaraza du 17 au 19 mai 2007. Wildlife Conservation Society , PN Virungas. Unpublished field report.

The forest elephant occurs in Kahuzi-Biega and in Maiko, two areas that consist mainly of tropical rainforest. The savanna elephant is found in Garamba, a savanna park and in Virunga which contains both forest and savanna habitats.

In the Kahuzi-Biega lowland forest, where the pre-war mean density of elephants was similar to that of the RFO [16], no signs of elephants were found 10 years later (Hart, J et al. Inventory and Monitoring report No 7, Nov 2007, Wildlife Conservation Society, DRC). Shortly after the beginning of the war, the area was occupied by rebel factions who were associated with the genocide in Rwanda. The park authorities (ICCN) quickly lost control over the area. Park guards were killed, equipment was stolen and infrastructure was damaged. Artisanal mines and settlements were established inside the park. Only in 2007 did ICCN regain control over part of the area. In the upland forest of the park, the situation was not much better and elephants declined from 800 to about 20 individuals. More than 150 elephant carcasses were found between 1997 and 2000.

Poaching in Maiko NP also affected elephants immensely (Table 5), and ivory helped fund the acquisition of arms at the start of the civil. As was the case with Kahuzi-Biega lowland forest, there was virtually no protection in place during this period. Compared to these two forest parks, more elephants survived in the RFO. This is very likely due to the fact that protection, although limited, was provided at a critical time when this was virtually non-existent in Kahuzi-Biega and Maiko.

In Garamba the decline in elephants was greater than in the RFO, but not as catastrophic as in Kahuzi-Biega and Maiko. Garamba's wildlife not only suffered during the civil conflict in DRC but it had already been affected from 1991 onward by the influx of 80,000 refugees escaping the civil war in Sudan and the installation of the SPLA (Sudan People's Liberation Army) near the park boundaries, which resulted in a sharp increase in bushmeat hunting. Illegal killing of elephants rose rapidly after 1996, when the DRC conflict began. SPLA rebels, militias hired by Mobutu, and later also the Congolese army (FARDC), were involved in poaching at different times [5]. Elephants concentrated in the south of the park, where most of the law enforcement patrolling occurred in different periods during this war.

In Virunga, only elephant populations in the savanna were monitored. There was ample evidence of illegal killing and elephants tended to keep to just a few large moving groups, presumably as a defense mechanism against poaching. However, the estimated population did not decline to the same extent as elsewhere in the country, probably because of elephants moving in from the adjacent Queen Elizabeth National Park in Uganda where they were well protected (Kujirakwinja D, Plumptre A, Moyer D, Mushemzi N. 2006. Parc National des Virunga. Recensement aerien des grands mammifères. Institut Congolais pour la Conservation de la Nature, Wildlife Conservation Society, US Fish and Wildlife Service; Andy Plumptre personal comments).

### Effects of the conflict on elephant distribution

The spatial patterns that we observed may be attributed to movements of elephants in response to hunting and insecurity that varied in space and time. The comparison between pre and post-war spatial models suggests that the role of humans in determining elephant distribution increased substantially during the war. Elephants have large home ranges and travel large distances. They often avoid areas of increased danger and can quickly move through areas of higher risk [17], [18]. Near the end and immediately after the conflict, elephants were more abundant in a core area in the south of the reserve. During the conflict, despite the overall decline, this area formed a refuge for elephants. Elephant hunting levels were lower there (Figure 2) and some protection was provided throughout the period of the worst elephant killing between 2002 and 2004. ICCN staff with support from the Wildlife Conservation Society (WCS), Gilman International Conservation (GIC), and UNESCO, deployed antipoaching patrols from headquarters in Epulu and two research zones (Lenda and Edoro, Figure 1). It has been shown that law enforcement is crucial for protecting large species [19] besides local social institutions that regulate hunting [20].

Several large-scale studies of multiple sites in Central Africa have shown a negative relationship between distance from roads and elephant densities, and this was often one of the best predictors of elephant distribution [21]–[25]. However, we did not find the same relationship in our study. Roads were not important in determining the pre-war distribution of elephants, and distance from the nearest road was a very weak predictor after the conflict. Thus, this relationship with roads is not always valid at the smaller spatial scale of an individual site, and may be confounded by other factors such as protection and habitat [26].

Ecological covariates such as habitat and slope did not contribute much in explaining elephant distribution, compared to human covariates. Some of this may be due to the coarse resolution that habitat types were sampled at in this study but, much more likely, humans had the overwhelming impact on elephants. In other places in Central Africa, elephants were more abundant in forests with dense herbaceous undergrowth, for example in secondary or logged forest [21], [23]. We did not find the same relationship. Elephants are also attracted to forest clearings that supply essential minerals [21]. Because we did not have a complete dataset of the small forest clearings (called “Edos”) that occur in the RFO, they were not included in the models.

### Impact of persistent factors and post-war recovery

Post-conflict dynamics can affect the recovery and restoration of wildlife. Research has shown that the impacts of conflict can persist long after the war ends; in particular, institutional changes, population movements and the availability of weapons can have long lasting negative impacts [27].

The future of the remaining elephant populations and their recovery in the RFO and DRC are being affected by persistent factors, alongside growing threats such as new road developments, growing human populations, immigration and continuing demands for bushmeat and other resources. The national human population growth rate in 2010 was 3.165% per annum (CIA, The World Factbook, https://www.cia.gov/library/publications/the-world-factbook/). The pressure on the remaining resources is further increased by new road constructions and the development of mining and forestry to supply international markets [28]. The availability of arms as a result of the war complicates protection. At the same time, institutional capacity and political support for conservation are woefully inadequate. Complicity of the authorities in poaching further hampers recovery of elephants and other species (Apopo CA. Rapport sur le braconnage à l'Eléphant et sur la commerce de l'ivoire dans et à la_périphérie de la Réserve de Faune à Okapis. Inventory and Monitoring Unit, Rapport No 3, December 2004, Widlife Conservation Society, Democratic Republic of Congo).

Wildlife will be among the first and most vulnerable of resources to disappear if DRC's resource conflicts are not resolved. The strengthening of institutions in charge of conservation and development, the curbing of illegal trade in ivory and bushmeat and the prioritization to protect national parks and wildlife by the government and international organizations, will be crucial to counter these growing threats [29], [30].

Having protected areas is not enough to save elephants in times of conflict. As expected, the war in the Democratic Republic of Congo had a large impact on elephant populations, including those in parks and reserves. However, despite massive declines in numbers, our study has shown that the commitment of highly motivated government field staff, and the continued support by international organizations to provide some protection on the ground, made a difference for their survival. In sites such as Kahuzi-Biega and Maiko, where this protection could not be provided, the losses were greater. Therefore, even limited efforts to invest in conservation during periods of political turmoil have benefits for biodiversity. There have been similar observations in Rwanda, where parks and reserves that received support from international NGO's were far less affected by the genocide of 1994 than sites with no support. Two elements were critical in the survival of these protected areas: first was the continued presence by committed staff, while second was the continued funding by international NGO's who did not suffer the same cutbacks in funding as did bilateral and multilateral agencies due to the conflict [3]. Unfortunately, many conservation projects follow a development aid model that is often cut off during times of political instability, as was also the case in DRC [29].

The fate of the remaining elephants will be determined by how the country and the international community deal with the aftermath of the war. They must respond to the existing threats and to new threats that result from growing human populations and increasing demand for natural resources.

## Materials and Methods

### Ethics statement

Relevant permission to conduct fieldwork was obtained from the Congolese authorities (Protocole d'accord, Accord de Siège entre la République Démocratique du Congo et la Wildlife Conservation Society, 3 avril 2003). No live animals were harmed or handled during the study.

### Study area

The Okapi Faunal Reserve is located in the Ituri forest in North-Eastern DRC between 1° and 2° 30′ N and 27° 30′ and 29° 30′ E and encompasses an area of 13700 km^2^ (Figure 1). It belongs to the North Eastern Congolian forest block [31]. More than 90% of the reserve is covered by dense tropical forest consisting of either humid mixed evergreen forest with dominant canopy species such as *Cynometra alexandri*, *Julbernardia seretii* and *Brachystegia laurentii*, or monodominant forest dominated by almost pure stands of *Gilbertiodendron dewevrei* (called Mbau). Mbau occurs in small patches to large blocks of several tens of square kilometers [32] and is found mainly in the southern and western parts of the reserve. Besides these two main vegetation types there are smaller patches of other types, such as swamp forest along rivers where there is poor drainage, and drier forest and shrub on granite inselbergs in the northern part of the reserve [33]. In the north, the forest borders a mosaic of forest-savanna. Secondary forest occurs mainly on abandoned agricultural clearings that were made by shifting cultivators in the vicinity of settlements.

A national road (RN4), connecting the east to the west of the country, bisects the southern half of the reserve. A second road runs along the eastern boundary of the reserve and a third one lies at some distance from its western boundary (Figure 1). Most stretches of the roads have been barely maintained since the 1960′s and are degraded. Along them are numerous small villages where people live on subsistence agriculture and hunting. Around these villages, small fields are cleared in secondary forest [31]. Four larger towns exist around the reserve (Mambasa, Niania, Wamba and Mungbere). The reserve headquarters, at Epulu, are located along the national road in the central part of the reserve. Besides these settled populations, the forest harbors some of the few remaining hunter-gatherer groups (Mbuti and Efe) in the world. These people hunt duikers (forest antelopes), primates and rodents, and also gather medicinal plants, tubers and other forest products [34].

The Okapi Faunal Reserve was created in May 1992 and was recognized as a World Heritage Site in December 1996. It contains possibly one of the largest remaining elephant populations in the country and numerous other forest mammals including Okapi (*Okapi johnstoni*), which is endemic to DR Congo [8].

### Mammal surveys

We compared animal density estimates from two surveys, one from before the conflict and one from after the conflict. The pre-war survey (further referred to as the “1995 survey”) covered the whole area of 13700 km^2^ and was carried out during several months between March 1994 and November 1995 by local field teams led by Dr. John Hart. The post-war survey (the “2006 survey”) was conducted under the WCS-Congo (Wildlife Conservation Society Congo) Inventory and Monitoring Unit program. This survey was necessarily conducted in two stages because of security concerns. The center of the reserve, north and south of the RN4, was surveyed between April and June 2005, and the remainder of the reserve was surveyed between November 2006 and May 2007 as circumstances permitted.

We used the same sampling design in the pre- and post-war surveys, comprising a total of 110 transects at 51 sampling locations. At each sampling location, one to maximum four transects were established in different compass directions, all starting from the same departure point. Transects were marked in the field during the first survey in 1995 and revisited in 2005 when GPS locations were also recorded. The length of each transect was between 2.5 to 5 km. Standard line transect methodology was employed to record all observations of mammals or their signs, such as dung and nests [35], [36]. A straight line was cut through the forest following a fixed compass bearing. Observers walked slowly on the transect line and used a hip-chain to measure the distance traveled. When an animal or an animal sign was detected at any distance from the transect line, the perpendicular distance from the transect line to each observation was measured to the nearest cm. The surveys were conducted by several teams, each team consisting of 5–6 people who had received similar training in wildlife survey and transect methodology. Several observers who took part in the pre-war surveys also participated in the post-war surveys.

Perpendicular distances were measured to dung of forest elephant *(Loxodonta africana cyclotis)* and other forest mammals. We also recorded nests of Chimpanzees (*Pan troglodytes*) and observations of other primates. In this article, only forest elephants are discussed.

### Spatial covariates

We predicted that patterns of elephant densities would be correlated with spatial covariates associated with humans and habitat. Formulating a-priori hypotheses guided the selection of covariates that we included in the spatial models (Table 1). We used proxies for human access (distance from roads), human presence (distance from all human settlements and from major towns only), protection (distance from park boundary and from park headquarters) and habitat (slope and ecozones).

Because human demographic data were not available for all of the survey periods, we used a composite “deforestation index” of non-forested land (mostly agriculture and urban development) and distance to non-forested land as an indicator for the extent and intensity of human activity. Forest cover probability maps were obtained for 1990 and 2000 from the Carpe Decadal Forest Change Mapping project (CARPE Decadal Forest Change Mapping (DFCM) Project, http://carpe.umd.edu/resources/dfcm) through Erik Lindquist from South Dakota State University. We constructed a grid with a grid cell size of a quarter degree by a quarter degree, and measured the area of non-forested land in 1990 and 2000 in each cell. This allowed us to quantify the amount of deforestation from 1990 to 2000 per grid cell. We clipped the grid to an area within a buffer zone of 15 km around the reserve. For each time period, we calculated a composite index representing the ‘deforestation environment’ at each transect based on distance from the transect to each cell of the grid and amount of deforestation in each cell:
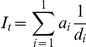



where 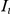 is the index at time t, 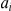 the extent of agriculture and other non-forested land in grid cell 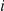, 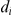 the distance from the middle of the transect to grid cell 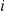. A high index represented large areas of deforestation (deforestation ‘hotspots’) close to the transect. We hypothesized that higher animal densities were correlated with a low index representing less human activity and vice versa.

### Data Analysis

#### Estimates of densities and changes in densities

Elephant dung densities were estimated, modeling the detection probability from the transect line, as described by Buckland et al. [37], using the software program DISTANCE 5.0 (Research Unit for Wildlife Population Assessment, http://www.ruwpa.st-and.ac.uk/distance/).

We used data from all locations in each time period to model the detection function. We explored several models that were available in DISTANCE (uniform, half-normal and hazard-rate) and selected the model with the best fit using Akaike's Information Criterion (AIC) [38]. To estimate densities, we grouped data from all transects within a sampling location to obtain independent sampling units.

To analyze changes in elephant densities between 1995 and 2006, we used a z-test to test the null hypothesis that the difference in density between both surveys was zero [37].

#### Spatial analysis and modeling

We used “Ordinary Kriging” in ArcGIS geostatistical analyst [39] (ESRI, http://www.esri.com/) to generate a continuous surface of elephant dung densities (Figure 3). This method is based on the assumption that objects closer to each other are more similar than objects further apart. Kriging assigns a weight to any particular point of a surface based on the measured values of neighbouring sampling locations, the distance to those locations and the overall spatial arrangement of the data points. We used this technique to inspect spatial patterns before modelling, and the maps also helped to interpret the results of the spatial models. We also performed a Getis-Ord Gi* hotspot analysis [40] to identify those transects where densities were higher or lower than expected by chance. This method compares the value of a local feature (in this case dung density on a transect) with neighbouring features and with the sum of all features. A local value that is significantly higher or lower than the expected local sum indicates a hotspot. For each transect we calculated a z-score and p-value for statistical significance. High z-scores indicate spatial clustering of either high (positive score) or low (negative score) values.

To test the a priori formulated hypotheses presented in Table 1, we developed spatial regression models using Generalized Additive Modeling (GAM). Hedley et al. [41] successfully used GAMs in combination with line transect sampling to model abundances of marine mammals as a function of spatial covariates. Similar techniques were later applied to model densities of elephants and other mammals by Blake et al. [22] and Stokes et al. [21].

We fitted density to spatial covariates in a GAM with the following form:
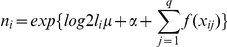



where 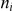 is the number of observations on transect 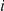, 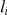 is the length of transect 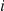, 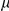 is the effective half-strip width (calculated in DISTANCE), 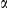 is the intercept and 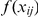 is a smooth function of covariate 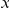 on the 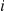 th transect. Density is modeled by including 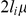 as an offset term which gives the effective area surveyed at transect 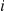.

Because the influence of a covariate is modelled using a smooth function instead of a linear function, as is the case with linear and generalized linear regression, the relationship between an independent and the dependent variable can include truly non-linear shapes that cannot be transformed into linear forms [42]. Errors showed signs of over-dispersion and we modelled these using a Quasipoisson distribution for over-dispersed data [43].

We examined multicollinearity between variables using scatterplots and Pearson correlation tests. We kept only those covariates with a correlation coefficient of less than 0.6, an arbitrary threshold that ensured that our a priori formulated hypotheses in terms of human presence, human access, protection and habitat were still represented. Distance from the nearest village was not included as a covariate because of its high linear correlation with distance from the nearest road.

We fited GAMs to single covariates and to a combination of covariates (composite models). For composite models, we used “mgcv” (multiple smoothing parameter estimation by Generalized Cross Validation) GAMs [44] in R (http://www.r-project.org), which provided automatic selection of smoothing parameters for each covariate using Generalized Cross Validation (GCV). “Mgcv” gams also give a good fit of data with many zeros if a Quasipoisson distribution is used [45]. Model simplification and model selection was carried out by the process of backward deletion. This involved starting with an initial model comprising all candidate covariates and then dropping terms sequentially. Each time a term was dropped, we checked plots and GCV scores (equivalent to AIC) to see if the deletion was warranted [44].

We compared spatial models for both time periods. We tested models with human-related covariates first and then models that included habitat covariates as well, to see if the latter could explain additional variation in density patterns.

## Supporting Information

Figure S1 **Conflict timeline. A Chronology of Military Occupation, Elephant Poaching, and ICCN Control in the RFO.** Year (first column), access to the reserve by park guards from ICCN (second column), intensity of elephant poaching (third column), conflict events (fourth column) and elephant related events (fifth column) in the RFO and region between 1996 and 2009.
(TIF)
