# Supplementary figures and images for: Resource Wars and Conflict Ivory: The Impact of Civil Conflict on Elephants in the Democratic Republic of Congo - The Case of the Okapi Reserve
Source: PLoS One. 2011 Nov 9;6(11):e27129. doi: 10.1371/journal.pone.0027129 (PMC3212536; doi:10.1371/journal.pone.0027129)

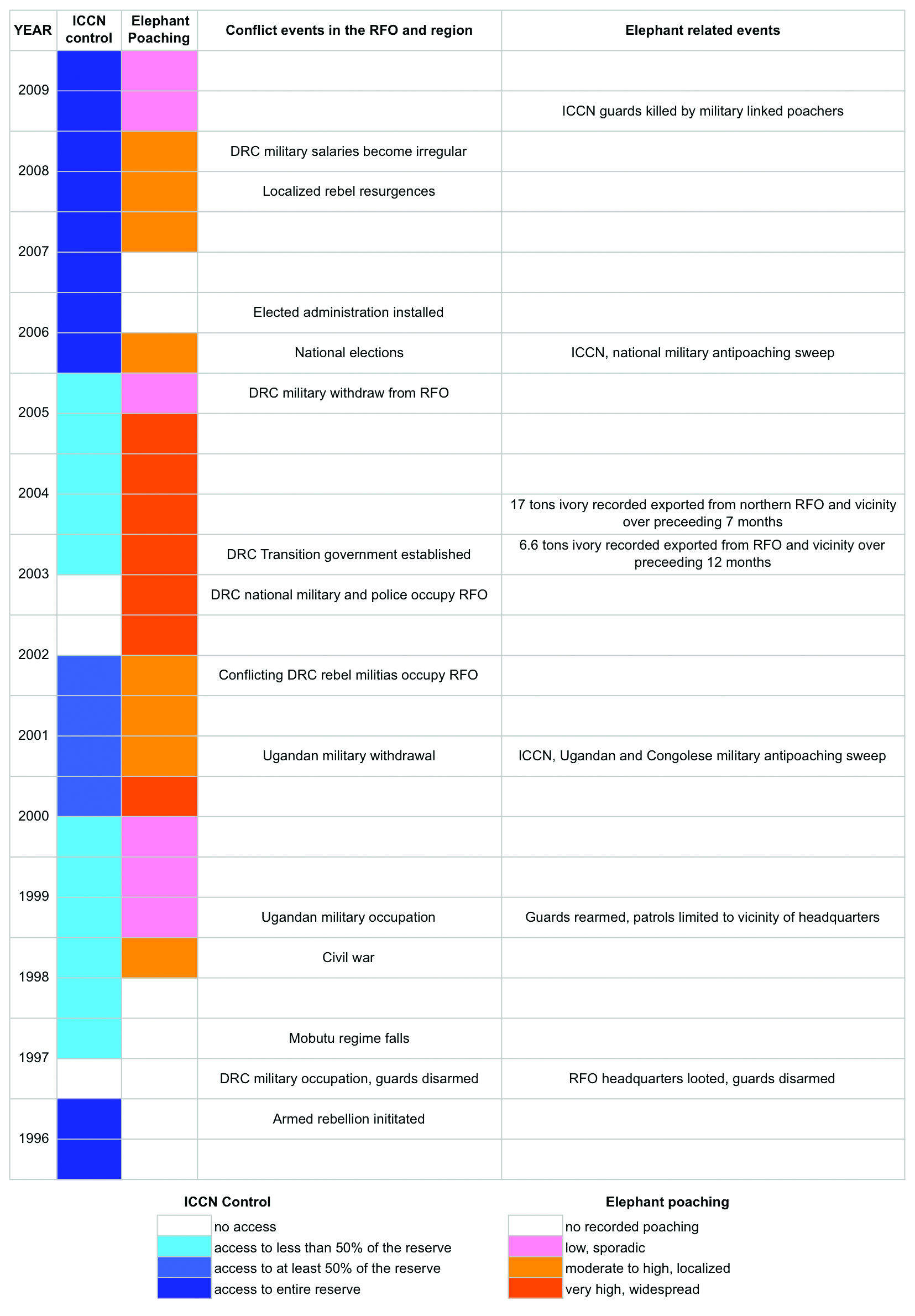

Supplement: Figure S1 — Conflict timeline. A Chronology of Military Occupation, Elephant Poaching, and ICCN Control in the RFO. Year (first column), access to the reserve by park guards from ICCN (second column), intensity of elephant poaching (third column), conflict events (fourth column) and elephant related events (fifth column) in the RFO and region between 1996 and 2009. (TIF) [file pone.0027129.s001.tif]
